# Comparison of Humoral and Cellular CMV Immunity in Patients Awaiting Kidney Transplantation

**DOI:** 10.3390/diagnostics11091688

**Published:** 2021-09-16

**Authors:** Monika Lindemann, Benjamin Wilde, Justa Friebus-Kardash, Anja Gäckler, Oliver Witzke, Ulf Dittmer, Peter A. Horn, Andreas Kribben, Nils Mülling, Ute Eisenberger

**Affiliations:** 1 Institute for Transfusion Medicine, University Hospital Essen, University of Duisburg-Essen, 45147 Essen, Germany; Peter.Horn@uk-essen.de; 2 Department of Nephrology, University Hospital Essen, University of Duisburg-Essen, 45147 Essen, Germany; Benjamin.Wilde@uk-essen.de (B.W.); Justa.Friebus-Kardash@uk-essen.de (J.F.-K.); Anja.Gaeckler@uk-essen.de (A.G.); Andreas.Kribben@uk-essen.de (A.K.); Nils.Muelling@uk-essen.de (N.M.); Ute.Eisenberger@uk-essen.de (U.E.); 3 West German Centre of Infectious Diseases, Department of Infectious Diseases, University Hospital Essen, University Essen-Duisburg, 45147 Essen, Germany; Oliver.Witzke@uk-essen.de; 4 Institute for Virology, University Hospital Essen, University Essen-Duisburg, 45147 Essen, Germany; Ulf.dittmer@uk-essen.de

**Keywords:** human cytomegalovirus, ELISpot, interferon-γ, dialysis, immunosuppressive therapy

## Abstract

Chronic kidney disease may alter antiviral T cell immunity. In the current study, we assessed in 63 patients prior to kidney transplantation how humoral and cellular immunity against cytomegalovirus (CMV) correlated using an interferon (IFN)-γ ELISpot (T-Track^®^ CMV, Mikrogen, Neuried, Germany). The cohort comprised 24 patients with negative and 39 with positive CMV IgG. Whereas none of the patients with negative CMV IgG showed detectable responses to the T-Track^®^ CMV, 26 out of 39 patients with positive CMV IgG had positive ELISpot responses. The median response to CMV pp65 in the CMV seronegative group was 0 spot forming units (SFU) per 200,000 PBMC (range 0–1) and in the seropositive group 43 SFU (range 0–750). Thus, 13 out of 39 patients with positive CMV serostatus (33%) had undetectable T cell immunity and may be at an increased risk of CMV reactivation. CMV pp65-specific ELISpot responses were 29.3-fold higher in seropositive patients with vs. without dialysis and 5.6-fold higher in patients with vs. without immunosuppressive therapy, but patients with dialysis and immunosuppressive therapy showed, as expected, lower responses to phytohemagglutinin, the positive control. This finding may be caused by (subclinical) CMV-DNAemia and a “booster” of CMV-specific T cells.

## 1. Introduction

In kidney transplant recipients, infection with herpesviruses, especially with cytomegalovirus (CMV), can lead to severe illnesses such as interstitial pneumonitis, hepatitis, colitis and encephalitis [1], and it can trigger allograft rejection [2]. In contrast, in immunocompetent individuals, the course of the infection is usually asymptomatic or mild, with symptoms similar to that of mononucleosis [3]. Thus, it is essential to assess the risk of CMV infection/reactivation as precisely as possible in transplant patients or patients awaiting transplantation. Apart from determining humoral immunity against CMV, i.e., immunoglobulin (Ig) G or M, the cellular immune response against CMV is increasingly assessed by in vitro methods, such as flow cytometry or the ELISpot [4]. Humoral immunity is known to be diminished in dialysis patients and/or under immunosuppression. For example, repeated examinations showed in 18 out of 168 hemodialyzed patients with CMV seropositivity (11%) a temporary change to negativity [5]. Furthermore, CMV-specific T cell immunity was detected in kidney transplant patients without CMV IgG antibodies [6]. A review by Sester et al. [7] emphasized the important role of CMV-specific T cell immunity in solid organ transplant recipients. However, it is also well established that impaired kidney function alters T cell function, leading to diminished antimicrobial responses [8]. Thus, despite previous CMV infection either specific antibodies or T cells may fall below a certain limit and become (temporarily) undetectable. The highly sensitive ELISpot assay can detect cytokine secretion on a single cell level [9], and it is feasible to measure antiviral immunity also in transplant recipients [10]. The quantification of cellular CMV immunity may help to stratify the risk of CMV infection or reactivation and thereby guide pre-emptive and prophylactic antiviral treatment after transplantation [11,12,13,14,15,16,17,18,19,20,21,22,23,24].

Specific immunity against CMV may not only be important in transplant recipients but also in patients awaiting kidney transplantation. On the waiting list, risk stratification of the patients—also considering CMV positivity/negativity—is performed. Impaired kidney function may influence cellular immune function, and there are few data on the correlation of humoral and cellular immunity in this cohort [25]. In the current study, we addressed the question how humoral and cellular CMV immunity correlated in 63 patients awaiting kidney transplantation. Furthermore, we analyzed if any covariate such as CMV IgG, CMV IgM, age, sex, cause of kidney failure, dialysis, prior kidney transplantation or immunosuppressive therapy had an influence on CMV-specific ELISpot responses.

## 2. Materials and Methods

### 2.1. Patients

From March to June 2021, we consecutively included 63 patients awaiting kidney transplantation (Table 1). There were no exclusion criteria. The group contained 38 males and 25 females; the median age was 54 years (range 19–78). Nineteen patients received peritoneal dialysis and 31 hemodialysis. Twelve patients were treated with immunosuppressive drugs. The study was approved by the local ethics committee of the University Hospital Essen, Germany (21-9883-BO), and all volunteers provided informed consent to participate in the study. The study was performed in accordance with the ethical standards noted in the 1964 Declaration of Helsinki and its later amendments or comparable ethical standards.

### 2.2. ELISpot Assay

Peripheral blood was collected in 9 mL heparin tubes, and the PBMC were isolated and adjusted to 2 million PBMC per milliliter. In total, 200,000 PBMC were added to each well of 8-well ELISpot strips and stimulated for 19 h at 37 °C in duplicates with two T-activated^®^ CMV proteins, immediate early antigen-1 (IE-1) and phosphoprotein 65 (pp65), according to the manufacturer’s instructions (T-Track^®^ CMV, Mikrogen GmbH, Neuried, Germany; formerly Lophius Biosciences GmbH, Regensburg, Germany) [10,26]. In parallel, negative controls (cells with medium only) and positive controls (stimulated with the mitogen phytohemagglutinin, PHA) were cultured. The resultant spots, each representing a single interferon (IFN)-γ releasing cell, were quantified using an ELISpot plate reader (AID Fluorospot, Autoimmun Diagnostika GmbH, Strassberg, Germany). The results were generated according to an algorithm provided by the manufacturer. Using the provided algorithm, the arithmetic mean of square-root-transformed 2-replicate spot counts is calculated and squared [26]. Negative controls were subtracted from CMV-specific values, resulting in spot forming units (SFU). SFU ≥ 10 were defined as positive.

### 2.3. CMV Serostatus

CMV IgG was determined using the Anti-CMV-IgG^®^ (DiaSorin, Saluggia, Italy) assay on the LIASON XL platform, following the manufacturer’s instructions. In addition, an Anti-CMV IgM^®^ (DiaSorin) was used to detect CMV primary infection. Using this platform, CMV IgG < 12 IU/mL is considered negative, from 12 to 14 IU/mL borderline and >14 IU/mL positive.

### 2.4. Statistical Analysis

Statistical analysis was performed with GraphPad Prism 8.0.1 (San Diego, CA, USA) and IBM SPSS Statistics 23 (New York, NY, USA) software. For the analysis of numerical variables, we applied Spearman correlation. To assess the impact of categorical covariates, we used Fisher’s exact test, Mann–Whitney or Kruskal–Wallis test as appropriate. We furthermore performed multivariate analysis using multinominal logistic regression. Here, we included age as continuous and CMV IgG and dialysis and immunosuppressive therapy as dichotomous variables, i.e., yes or no. Two-sided *p* values < 0.05 were considered significant.

## 3. Results

**Figure 1:**
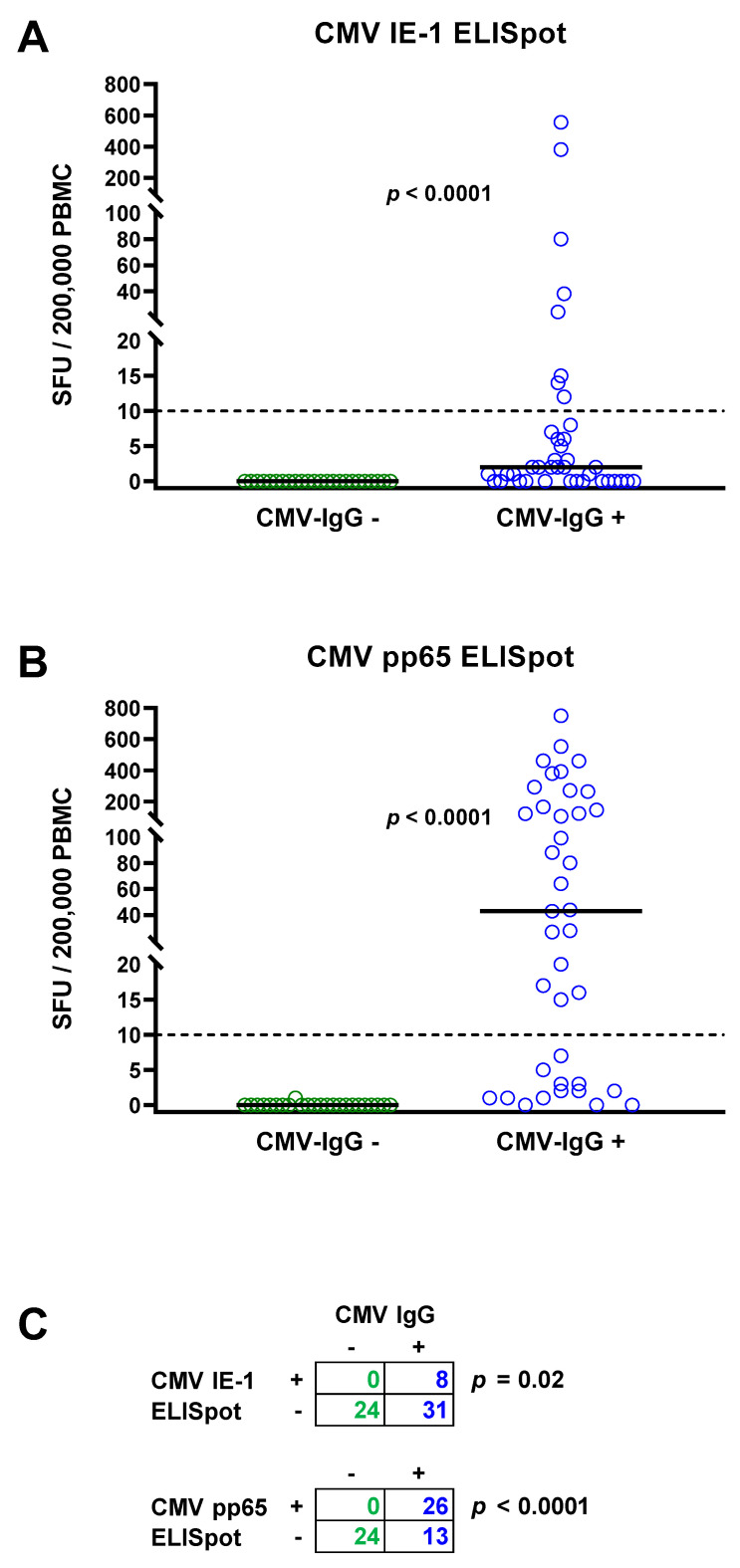
Distribution of CMV-specific ELISpot responses in 63 patients awaiting kidney transplantation. The cohort comprised 24 with negative (green circles) and 39 with positive CMV IgG (blue circles). Panel (**A**) shows ELISpot responses upon stimulation with CMV immediate early antigen 1 (IE-1) and panel (**B**) upon stimulation with CMV phosphoprotein 65 (pp65). Horizontal lines indicate median values. The dotted line represents the cutoff for positive responses (10 spot forming units, SFU). Cellular in vitro responses in patients without and with CMV IgG were compared by Mann–Whitney test. Panel (**C**) summarizes the correlation between humoral (IgG) and cellular (ELISpot) CMV-specific responses (Fisher’s exact test).

The total cohort of 63 patients with chronic kidney disease consisted of 24 patients with negative and 39 with positive CMV IgG. None of the patients with negative CMV IgG showed detectable IFN-γ responses to the CMV IE-1 ELISpot (Figure 1A) or to the CMV pp65 ELISpot (Figure 1B). The median response to the CMV IE-1 protein was 0 SFU per 200,000 PBMC (range 0–0) and to the CMV pp65 protein 0 SFU (range 0–1), respectively (Figure 1A,B, Table 2). In patients with positive CMV IgG, cellular responses were significantly higher (*p* < 0.0001). The median response to CMV IE-1 was 2 SFU (range 0–555), and to CMV pp65, it was 43 SFU (range 0–750). Fisher´s exact test showed that ELISpot responses to CMV IE-1 and to pp65 were significantly (*p* < 0.05) correlated with CMV IgG responses (Figure 1C).

**Figure 2:**
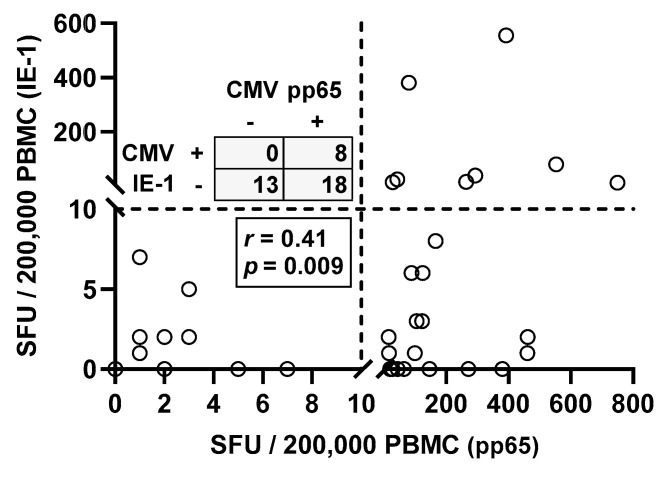
Spearman correlation analysis of T cell immunity toward CMV IE-1 and pp65 in CMV IgG positive patients awaiting kidney transplantation (n = 39). The dotted lines represent the cutoff for positive responses (10 spot forming units, SFU) for each of the CMV antigens. Quadrant statistics is shown in the upper left panel and results of the Spearman correlation analysis in the lower left panel.

Moreover, Spearman correlation analysis of responses toward the two CMV proteins IE-1 and pp65 in CMV seropositive patients showed, as expected, positive correlation (*r* = 0.41, *p* = 0.009) (Figure 2). Thirteen out of 39 patients with positive CMV IgG (33%) showed negative ELISpot responses to both CMV antigens. Eighteen patients responded only to CMV pp65 but not to IE-1 and eight responded to both antigens.

Taken together, only 67% of patients with positive CMV serostatus had detectable T cell immunity, i.e., only 26 out of 39 patients with positive CMV IgG also had positive responses to the T-Track^®^ CMV. All these patients had a positive response to CMV pp65 and eight a positive response to IE-1 and pp65.

**Figure 3:**
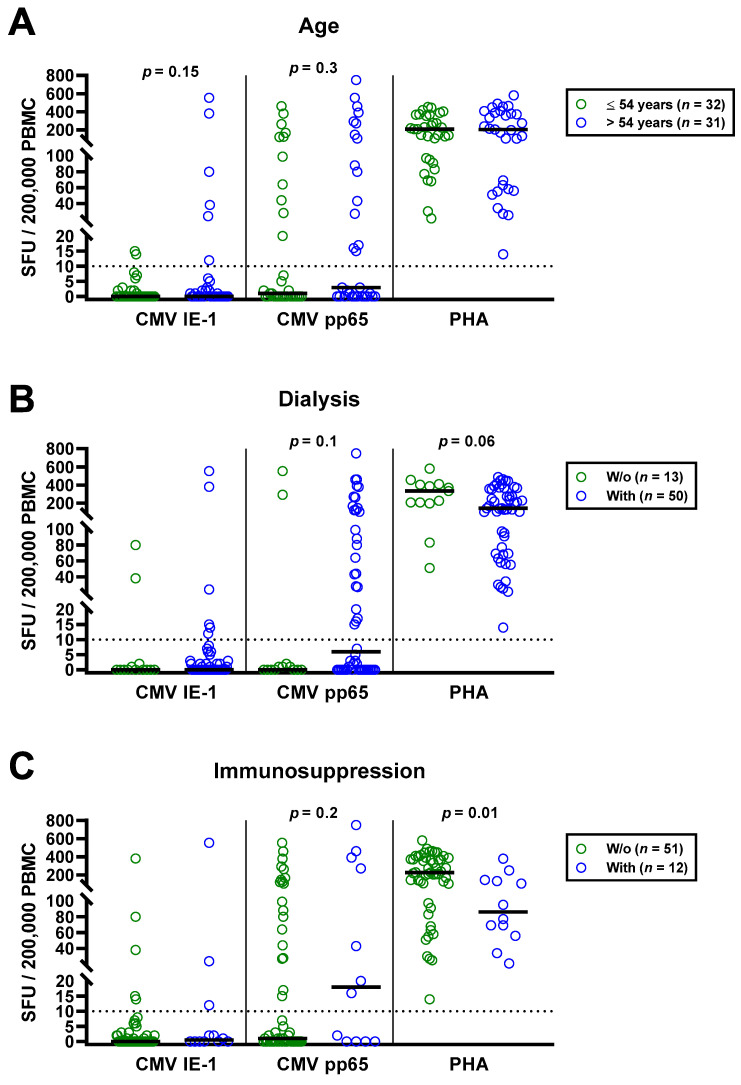

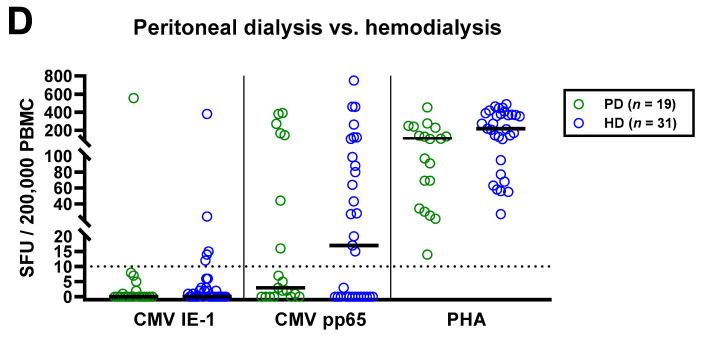
Distribution of CMV-specific ELISpot responses in 63 patients awaiting kidney transplantation, divided either by age (**A**), presence or absence of dialysis (**B**), immunosuppression (**C**) or peritoneal dialysis vs. hemodialysis (**D**). Horizontal lines indicate median values. The dotted line represents the cutoff for positive responses (10 spot forming units, SFU). Cellular in vitro responses toward CMV immediate early antigen 1 (IE-1), CMV phosphoprotein 65 (pp65) and the positive control phytohemagglutinin (PHA) were compared by Mann–Whitney test. PD, peritoneal dialysis; HD, hemodialysis.

The univariate analysis for potential covariates in 63 patients awaiting kidney transplantation indicated that none had a significant impact on CMV-specific ELISpot responses, except for CMV IgG. Spearman correlation analysis showed no correlation with age (IE-1: *r* = 0.02, *p* = 0.9; pp65: *r* = 0.16, *p* = 0.2). We furthermore split the cohort into younger and older individuals, divided by the median age (54 years), and compared both by Mann—Whitney test (Figure 3A). Although CMV-specific T cell responses tended to be higher in older patients, the difference escaped statistical significance (IE-1: *p* = 0.15; pp65: *p* = 0.30). In younger vs. older patients, we observed median responses of 0 vs. 0 SFU toward IE-1 and 1 vs. 3 SFU toward pp65. The respective mean values were 1.9 vs. 35.9 and 55.9 vs. 105.4 SFU. Mann–Whitney test also yielded no significant influence of CMV IgM, sex and presence or absence of dialysis, prior kidney transplantation or immunosuppressive therapy (Table 2). Finally, Kruskal–Wallis test indicated that the cause of kidney failure or the number of previous transplantations had no significant impact.

The impact of dialysis and immunosuppressive therapy on CMV-pp65 specific responses escaped statistical significance (*p* = 0.1 and *p* = 0.2, respectively) (Figure 3B,C). Whereas CMV-pp65 specific responses in patients with dialysis and immunosuppression were higher, we observed the opposite for the positive control with PHA. This difference for the PHA control was significant in patients with vs. without immunosuppression (*p* = 0.01). Furthermore, median responses to CMV pp65 tended to be lower in 19 patients with peritoneal dialysis than in 31 hemodialysis patients (3 vs. 17 SFU, Figure 3D). Detailed data on the twelve patients who received immunosuppressive therapy are presented as Table 3. Two patients who received T cell depletion with anti-thymocyte globulin more than five years ago both showed detectable T cell responses to CMV pp65. Moreover, CMV pp65-specific responses were observed in all patients with prior liver transplantation (*n* = 2) and with 2–3 prior kidney transplantations (*n* = 3).

We also performed multivariate analysis, which included CMV IgG, age, dialysis and immunosuppressive therapy. We found significant results for the following variables: IE-1 ELISpot: CMV IgG (*p* = 0.01); pp65 ELISpot: CMV IgG (*p* < 0.0001), age (*p* < 0.0001) and immunosuppressive therapy (*p* = 0.048).

**Figure 4:**
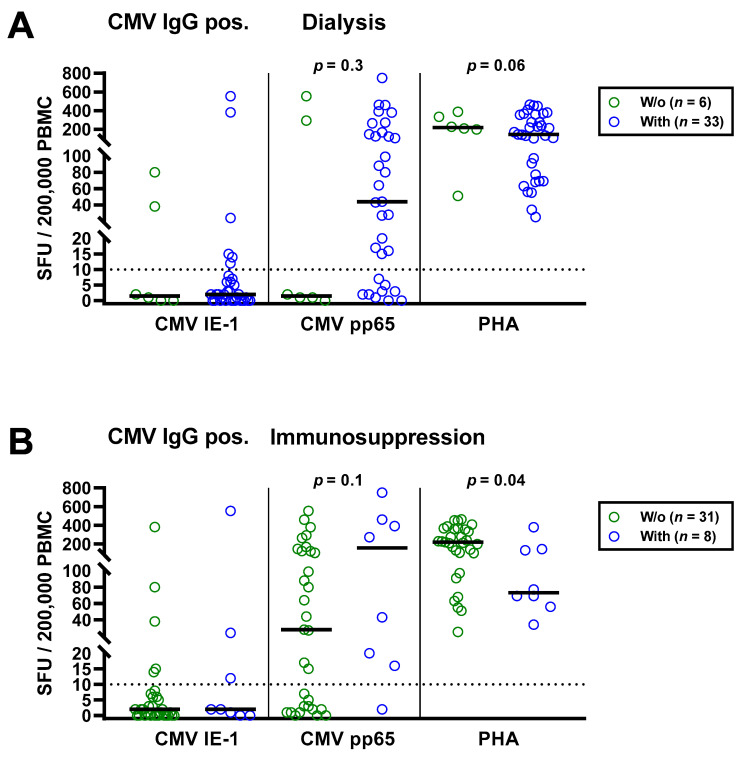
Distribution of CMV-specific ELISpot responses in 39 CMV IgG positive patients awaiting kidney transplantation, divided either by presence or absence of dialysis (**A**) or immunosuppression (**B**). Horizontal lines indicate median values. The dotted line represents the cutoff for positive responses (10 spot forming units, SFU). Cellular in vitro responses toward CMV immediate early antigen 1 (IE-1), CMV phosphoprotein 65 (pp65) and the positive control phytohemagglutinin (PHA) were compared by Mann–Whitney test.

As a next step, we separately analyzed the subgroup of 39 patients with positive CMV IgG (Table 4). Similar to the total cohort, CMV-specific ELISpot responses were stronger in the patients with dialysis and with immunosuppressive therapy (Figure 4). CMV pp65 specific SFU were 29.3-fold higher in patients with vs. without dialysis (median of 44 vs. 1.5) and 5.6-fold higher in patients with vs. without immunosuppressive therapy (median of 157 vs. 28), respectively. However, this finding was also non-significant. Again, PHA controls showed the opposite of CMV pp65 stimulated cultures, a diminished response in patients with dialysis or immunosuppression.

## 4. Discussion

In this study, we tested 63 patients with chronic kidney disease for their correlation of humoral and cellular CMV immunity. We observed that the group of patients with CMV IgG seropositivity can be divided into one-third without detectable cellular immunity and two thirds with cellular immunity. Cellular responses to the CMV pp65 proteins were considerably more abundant than responses to CMV IE-1, which could be detected in 26 and eight out of 39 CMV IgG seropositive patients, respectively. In patients with CMV IgG seropositivity but with undetectable T cell responses, antiviral prophylaxis after kidney transplantation may be a suitable therapeutic option.

To better assess the strength of the ELISpot responses in the current cohort, we compared it with results of kidney and liver transplant recipients from our center, whose data have been partially published [10,26]. Both previous studies included intermediate-risk (donor (D)−/recipient (R)+, D+/R+) and high-risk (D+/R−) transplant recipients. The strength of ELISpot responses toward the T-Track^®^ CMV in kidney transplant recipients and in liver was similar to the current data. In kidney transplant recipients, median CMV IE-1 specific responses of 1.5 SFU per 200,000 lymphocytes and median CMV pp65 specific responses of 40.5 SFU per 200,000 lymphocytes were detected (*n* = 22) [10]. In liver transplant recipients, we found median CMV IE-1 specific responses of 4 SFU per 200,000 lymphocytes and median CMV pp65 specific responses of 41 SFU per 200,000 lymphocytes (*n* = 103) [26]. When normalizing our current data on PBMC to lymphocytes and applying a conversion factor of 1.29 as calculated from raw data of the later study by Gliga et al. [26], responses were still at a similar level (converted data of the current study: CMV IE-1 and pp65: 3 and 55 SFU per 200,000 lymphocytes, respectively).

As compared to a previous study on hemodialysis patients [25], the rate of positive ELISpot responses in CMV seropositive patients was lower in the current study. The study by Banas et al. observed positive T-Track^®^ CMV results in 90% (60/67) of CMV-seropositive hemodialysis patients. Moreover, they detected IE-1-reactive cells in blood samples of patients with a negative CMV serology. This previous study observed median CMV IE-1 specific responses of 9.7 SFU per 200,000 lymphocytes and median CMV pp65 specific responses of 165 SFU per 200,000 lymphocytes, respectively. It is possible that laboratory-dependent differences, e.g., in the ELISA used to categorize patients or in the definition of spots, may be a reason for the different results. The previous study used another ELISpot reader than ours, which is known to have a major impact on spot numbers.

Furthermore, in a multicenter study on 96 intermediate-risk (D−/R+, D+/R+) renal transplant recipients also tested by T-Track^®^ CMV [11], CMV-specific ELISpot responses were higher. In the study by Banas et al., median CMV IE-1 specific responses of 9–25 SFU per 200,000 lymphocytes and median CMV pp65 specific responses of 114–206 per 200,000 lymphocytes were detected, depending on the time point. Thus, spot numbers measured by the group of Banas et al. seem to be generally higher, either in hemodialysis patients or kidney transplant recipients. This difference makes clear that cutoff values must always be validated by the respective laboratory. We presumably have defined only the patients with strong T cell responses as positive, which are more likely protected from CMV reactivation.

In addition, the analysis of covariates showed that CMV IgG was significantly correlated with CMV-specific ELISpot responses. Furthermore, dialysis and immunosuppressive therapy may both affect cellular CMV-specific immunity. CMV seropositive patients receiving dialysis or immunosuppressive treatment had 29.3-fold or 5.6-fold higher responses toward CMV pp65 than those without dialysis (i.e., patients on the waiting list prior to living kidney transplantation) or without immunosuppressive treatment. In contrast, PHA responses were diminished in patients with dialysis or immunosuppression. This finding on CMV immunity is unexpected at first sight since both dialysis and immunosuppression should lead to impaired T cell function. However, immunosuppression was found, as expected, after stimulation with the mitogen PHA. These data are consistent with a previous study by our group showing that patients with multiple myeloma had impaired T cell responses to a wide range of mitogens and recall antigens but not to CMV [27]. In detail, T cell responses toward the four mitogens PHA, concanavalin A, pokeweed mitogen and anti-CD3 and the seven recall antigens tuberculin, tetanus toxoid, *Candida albicans*, herpes simplex virus-type 1, varicella zoster virus and influenza A and B virus were significantly lower in patients with multiple myeloma (*n* = 169) than in matched healthy controls (*n* = 100). However, T cell responses toward CMV did not differ significantly, and the percentage of positive CMV-specific responses in the patients was significantly higher than in the controls.

Taken together, the current data indicate that CMV-specific cellular immunity may be preserved or increased despite general suppression of T cell immunity (PHA response). Furthermore, our study in another immunocompromised cohort (patients with multiple myeloma) [27] suggests that immunosuppression does not affect all antimicrobial immune responses equally. 

Previous studies indicate that age significantly affects an individual’s immune response toward CMV [26,28,29]. The current study could confirm these findings, when performing multivariate analysis. In addition to age, dialysis (especially hemodialysis) affected cellular CMV immunity. A previous study has shown that hemodialysis leads to more pronounced inflammation compared to peritoneal dialysis, which may cause premature aging of the immune system [30]. Moreover, CMV infection plays a major role in immunological aging of patients with end-stage renal disease [31].

In conclusion, in CMV-seropositive patients with chronic kidney disease, a CMV pp65 specific ELISpot may determine the susceptibility to CMV reactivation. In one-third of these patients, an increased risk is assumed due to the absence of specific T cell responses. CMV-specific spot numbers were increased in patients receiving dialysis and immunosuppressive therapy, which may correlate with (subclinical) CMV-DNAemia and a “booster” of CMV-specific T cells. However, this hypothesis needs to be confirmed in larger, independent studies.

## Figures and Tables

**Table 1:** Characteristics of 63 patients awaiting kidney transplantation.

Variable	Group	Absolute Number or Median (Range)
Sex	Male	38
	Female	25
Age (years)		54 (19–78)
Renal disease	Diabetic glomerulosclerosis	8
	Chronic glomerulonephritis	16
	Nephrosclerosis	11
	Polycystic kidney disease	6
	Tubulointerstitial nephritis	4
	Congenital anomalies	1
	Autoimmune disease	4
	Amyloidosis	1
	Reflux nephropathy	1
	Thrombotic microangiopathy	0
	Other/not specified	11
Patients on dialysis		50
Time on dialysis (months)		19 (1–175)
Peritoneal dialysis		19
Hemodialysis		31
Preemptive patients		13
eGFR (mL/min/1.73 m²)		10.8 (3.8–23)
Prior kidney transplantation		7
Prior liver transplantation		2
Immunosuppressive drugs	CNI	6
	Steroids	9
	MPA	3
	ATG	2
	Azathioprine	2
	Hydroxychloroquine	1

Age, time on dialysis and eGFR are given as median and range. Overall, 12 patients received immunosuppressive therapy (5 with mono-therapy; 7 with combined therapy). eGFR, estimated glomerular filtration rate (only given for preemptively listed patients); CNI, calcineurin inhibitors; MPA, mycophenolic acid; ATG, anti-thymocyte globulin.

**Table 2:** Potential covariates of CMV-specific ELISpot responses in all patients awaiting kidney transplantation (*n* = 63).

Covariate	Group	CMV IE-1 ^1^ Median (Range)	CMV pp65 ^1^ Median (Range)
CMV IgG	Negative (*n* = 24)	0 (0–0)	0 (0–1)
	Positive (*n* = 39)	2 (0–555)	43 (0–750)
	*p*	**<0.0001**	**<0.0001**
CMV IgM	Negative (*n* = 61)	0 (0–555)	2 (0–750)
	Positive ^2^ (*n* = 2)	2 (2–2)	231 (1–461)
	*p*	0.2	0.3
Sex	Male (*n* = 38)	0 (0–381)	3 (0–750)
	Female (*n* = 25)	0 (0–555)	1 (0–392)
	*p*	0.9	0.6
Dialysis	No (*n* = 13)	0 (0–80)	0 (0–553)
	Yes (*n* = 50)	0 (0–555)	6 (0–750)
	*p*	0.5	**0.1**
Prior kidney transplantation	No (*n* = 7)	0 (0–555)	2 (0–553)
	Yes (*n* = 56)	0 (0–24)	2 (0–750)
	*p*	0.7	0.9
Immunosuppressive therapy	No (*n* = 51)	0 (0–381)	1 (0–553)
	Yes (*n* = 12)	0.5 (0–555)	18 (0–750)
	*p*	0.4	**0.2**

^1^ ELISpot responses are given as spot forming units (SFU) and the responses of two groups were compared by Mann–Whitney test; of which the *p* value is indicated. ^2^ One of these CMV IgM results was borderline. Results considered most relevant are marked with bold *p* values.

**Table 3:** Characteristics of twelve patients awaiting kidney transplantation who received immunosuppressive therapy.

No.	Sex	Age	Dialysis	Prior Tx	CNI	Steroids ^1^	MPA	ATG	Others	CMV PCR	CMV IgG	CMV IE-1	CMV pp65
					(ng/mL)	(mg)	(mg)			(IU/mL)		(SFU)	(SFU)
1	m	52	HD	1 LTx	6.7	-	2000	-	-	<65	1	2	461
2	f	56	PD	1 LTx	6.7	-	-	-	-	<65	1	1	16
3	m	46	HD	3 KTx	4	10	-	01/2016	-	n.t.	1	0	20
4	f	55	HD	2 KTx	4	2.5	-	-	-	n.t.	1	24	43
5	m	55	HD	2 KTx		5	-	01/2007	-	<65	1	12	750
6	f	32	PD	1 KTx	3	5	1000	-	-	n.t.	1	2	2
7	m	21	PD	1 KTx	4	5	1000	-	-	<65	0	0	0
8	m	49	PD	1 KTx	-	5	-	-	-	n.t.	0	0	0
9	f	40	HD	1 KTx	-	5	-	-	-	n.t.	0	0	0
10	m	61	PD	-	-	2.5	-	-	100 mg AZA	n.t.	0	0	0
11	f	69	PD	-	-	2.5	-	-	100 mg AZA	n.t.	1	0	271
12	f	63	PD	-	-	-	-	-	400 mg HCQ	n.t.	1	555	392

^1^ Prednisolone. CNI, calcineurin inhibitors; MPA, mycophenolic acid; ATG, Anti-thymocyte globulin; IE-1, immediate early antigen 1; pp65, phosphoprotein 65; SFU, spot forming units; HD, hemodialysis; PD, peritoneal dialysis; LTx, liver transplantation; KTx, kidney transplantation; AZA, azathioprine; HCQ, hydroxychloroquine (due to systemic lupus erythematosus); n.t., not tested. Positive antibody or T cell results are highlighted red.

**Table 4:** Potential covariates of CMV-specific ELISpot responses in CMV IgG positive patients awaiting kidney transplantation (*n* = 39).

Covariate	Group	CMV IE-1 ^1^ Median (Range)	CMV pp65 ^1^ Median (Range)
Dialysis	No (*n* = 6)	1.5 (0–80)	1.5 (0–553)
	Yes (*n* = 33)	2 (0–555)	44 (0–750)
	*p*	0.8	**0.3**
Immunosuppressive therapy	No (n = 31)	2 (0–381)	28 (0–553)
	Yes (n = 8)	2 (0–555)	157 (2–750)
	*p*	0.5	**0.1**

^1^ ELISpot responses are given as spot forming units (SFU) and the responses of two groups were compared by Mann–Whitney test, of which the *p* value is indicated. Results considered most relevant are marked with bold *p* values.

## Data Availability

The data can be obtained from M.L. upon request.
